# Progress and Challenges in Ex Situ Conservation of Forage Germplasm: Grasses, Herbaceous Legumes and Fodder Trees

**DOI:** 10.3390/plants9040446

**Published:** 2020-04-02

**Authors:** Jean Hanson, Richard H. Ellis

**Affiliations:** 1 International Livestock Research Institute, P.O. Box 5689, Addis Ababa, Ethiopia; 2 School of Agriculture, Policy and Development, University of Reading, PO Box 237, Reading RG6 6AR, UK; r.h.ellis@reading.ac.uk

**Keywords:** genebanks, forage germplasm, grasses, legumes, seed storage, conservation, seed longevity, seed germination, monitoring, regeneration

## Abstract

Forages provide an important livestock feed resource globally, particularly for millions of smallholder farmers, and have important roles in natural resource management and carbon sequestration, reducing soil erosion and mitigating the effects of climate change. Forage germplasm remains the basis for the selection and development of new, higher-yielding and better adaptedgenotypes to meet the increasing demand for livestock feed. Rapid rates of genetic erosion of forage diversity due to land-use change from natural pastures and rangelands to crop production to meet the food security requirements of a growing global population, together with pressures from a changing climate, highlight the necessity for ex situ seed conservation of forage genetic resources to provide germplasm for use by future generations. Whilst many forage species have orthodox seeds, the diverse range of genera and species which provide forage is a challenge in terms of the wide scope of information and understanding on conservation methods that genebank managers require—particularly for tropical forages, many of which are comparatively under-researched. We review the challenges to the conservation of tropical forage species by seed in ex situ genebanks and provide information on optimum methods for their management.

## 1. Introduction

The term ‘forage’ encompasses many different types of plants used for livestock feed, including grasses, herbaceous and tree legumes, as well as other non-leguminous forbs and trees varying greatly in plant habit and adaptation. Forages are an important livestock feed resource globally, particularly for millions of smallholder farmers who rely on natural pastures and grasslands as the basis of their sustainable livestock systems, allowing ruminants to convert feed that cannot be used directly by humans into milk and meat to provide essential nutrients that are required for human health, growth and cognitive development [1]; these animals also provide fibre and skin for clothing and footwear. Forages are effective in maintaining the natural resource base [2]. They stabilise the soil, provide ground cover and windbreaks to prevent or reduce soil erosion, increase soil carbon content by strong rooting and decomposition of leaf litter, and, through symbiotic nitrogen fixation by rhizobia with legumes, they capture nitrogen from the atmosphere to the soil. There has been much research aimed at capturing these benefits, and there are now many alternative ways of introducing sown forages into the farming system [2].

Most forage diversity originates in natural grasslands, one of the largest and most important natural ecosystems [3] covering over 52 million square kilometers globally. Grasslands are home to over a thousand forage species with actual or potential use to support livestock production systems in tropical and sub-tropical areas, although only about 70 species have been commercially developed, mostly through the direct selection of germplasm accessions or by plant breeding in few important species, as feeds in these regions (Table 1). Rapid rates of genetic erosion of forage diversity are occurring in grasslands, mainly due to land-use change converting natural pasture and marginal areas to crop production to meet the food security requirements of a growing global population. This erosion together with pressures from a changing climate, resulting in extreme weather events (such as drought, floods and cyclones), has a negative effect on natural habitats where forage diversity is still found and highlights the necessity for ex situ conservation of forage genetic resources to provide diverse forage germplasm for the use of current and future generations. Most forages in use today are wild species or genotypes selected from wild populations and safeguarding sources of forage germplasm is particularly important because with few forage breeding programmes [4]—and most of those focusing mainly on temperate grassland species, including alfalfa, ryegrasses and clovers—forage germplasm remains the basis for the future selection and development of the new feeds which are particularly needed for tropical and sub-tropical regions. 

**Figure 1:**
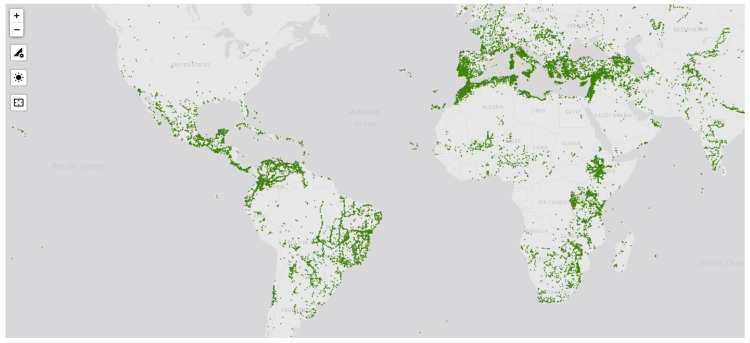
Map of forage accession collection sites. Data accessed through Genesys Global Portal on Plant Genetic Resources, www.genesys-pgr.org, 12 February 2020.

Forages are not as well represented as food crops in ex situ collections with almost 182,000 accessions representing over 1000 species of grasses, legumes and fodder trees maintained in 80 national and international genebanks registered in Genesys (www.genesys-pgr.org) compared to the approximately 7.4 million plant accessions stored in around 1750 genebanks globally [5]. These accessions registered in Genesys were collected from a wide range of sites across tropical and temperate regions (Figure 1) and contain a large amount of diversity with potential use in forage breeding. However, despite the diversity available in ex situ collections, the germplasm from these genebanks remains underutilised in crop and forage breeding programmes. Current genomic technologies that can be used to screen large collections efficiently offer opportunities for increased identification of useful genes and the use of germplasm [5].

## 2. Management of Forage Germplasm

Seed genebank protocols have been developed with both crop and wild species in mind, but the focus of most genebanks is very much crop genetic resources conservation with an acknowledgement of the priority to include crop wild relatives in collections. Whilst the knowledge base and dominant thinking was around conservation of crop plants, the same principles have been applied successfully to the ex situ conservation of wild plants [6,7]. The principle difference, and source of problems in implementing standard protocols, is that seeds from wild populations, crop wild relatives and forages tend not to show the high intra-seed lot uniformity of crop seeds [7]. Seed of forage species show considerable variability within and between seed lots and, similarly, within and between accessions of a species that make it difficult to develop inclusive protocols.

In the case of forage species, the information available on seed handling from seed collection through storage to germination, multiplication and regeneration is less than for the major staple food crops, and many of the problems facing forage plant genebank managers are similar to those facing those managing wild species genebanks:

- A wide range of diverse species;
- High variability amongst and within each species;
- Comparatively little relevant published information for many species;
- and limited practical experience with some species.

Nonetheless, it is wrong to imply that little is known about seeds of forages: Table 2 summarises examples of selected sources of information on protocols and advice on seed production, dormancy, germination, and survival in genebank storage for various genera of forage grasses and forage legumes. The International Seed Testing Association (ISTA) has included germination test methods for many forage species in the recent rules for seed testing [8]. Detailed information on seed longevity of forages is also included in several publications [9,10,11,12]. Even for species or genera in which advice cannot be found, such information sources are useful to indicate how to handle seed of similar species or genera. Nonetheless, some of this information and advice was developed for commercial seed production and may need to be interpreted and adapted for use by genebanks to support the ex situ conservation of forage genetic resources. Some of the particular difficulties in handling forage seed accessions in genebanks stem from the tendency of forage species to show substantial within-accession variation in the timing of flowering, seed development and maturation; they reflect plant diversity in general. As a result, few generalisations are possible when discussing the production, harvesting, and pre-storage processing of seeds of forage species. These diverse species can be determinate or indeterminate in their development; annual, biennial, or perennial; flowers can be self-fertile, require cross-pollination or are apomictic, or a mixture of the three; and flowering may often continue for several weeks [13,14,15,16,17,18,19]. Moreover, the spike in most grasses is fragile, with abscission at the rachis or rachilla, and thus, seeds shed to the ground as they mature [20]. Once mature, the seeds also differ in dormancy, germination requirements and in survival during storage [21].

## 3. Germplasm Collecting

One major difference between cultivated and wild forages and the major food crops is the collection location and strategy. Wild forages are found in grasslands and natural pastures, either with small numbers of plants distributed over a large area or as large areas of single species in open grasslands, compared to crop germplasm, which is found as landraces in farmers’ fields. Whilst crop landraces have more uniform maturity and little seed shattering, forages tend to vary within accessions in the timing of seed maturity, and in some grasses, seed shattering occurs as seeds ripen or pods dehisce in some forage legumes. This results in some seeds being collected whilst they are immature and unable to survive the drying and storage process, resulting in poor quality or small numbers of seeds being stored [6]. In some species, a post-harvest ripening treatment can be applied, keeping seeds in high humidity to facilitate ripening [22]. With only small numbers of seeds being collected for individual accessions, seed multiplication is usually required before conservation compared to crop landraces, which can often be collected in the required quantity of seeds to go straight for conservation, safety duplication and distribution. In some cases where the species covers large areas, such as in native grasslands, random collection strategies can be applied similar to those used for crops. Some forages must be collected as cuttings because at collection time there are few ripe seeds due to shattering, as reported in the germplasm of *Panicum coloratum* [23], or the species is a poor seeder and only a small percentage of the florets result in caryopses, as in Napier grass (*Pennisetum purpureum*) [24].

## 4. Multiplication and Regeneration

Regeneration and multiplication are especially challenging for forages; it can be a slow process for perennial species and accessions represented by few seeds. As explained above, the original sample size of many accessions is small, and most need to be multiplied at the outset to provide sufficient seeds for long term storage. In the absence of information on the amount of outcrossing in many forage species, each accession should be assumed to be fully outcrossing and thus isolated to ensure the maintenance of genetic integrity. In addition, there is a high risk of loss of diversity and changes in genetic integrity due to the small sample size available to use for the regeneration of accessions with few seeds [25]. Accessions of slow-growing and maturing fodder trees often remain in the field for several years before they are capable of flowering to produce enough seeds to meet storage, distribution and safety duplication needs. This results in limited availability of accessions of some species. General guidelines for regeneration procedures for forages have been developed for grasses and forage legumes [26,27]. 

Protocols for large-scale seed production and multiplication and the basic problems inherent in forage seed multiplication, collection, and regeneration are similar but not identical. Many forages are recognised as crops, but they tend to be selected for vegetative rather than reproductive traits. Thus selection for long periods of vigorous vegetative growth by forage plant breeders to benefit livestock production has had to be tempered by the requirement for the plants to ultimately flower and produce sufficient seeds if new varieties are to be multiplied and disseminated [28]. Moreover, many forages have not been subject to plant breeding, which has focused on *Medicago* species, clovers, ryegrasses, oats, *Brachiaria* grass, Guinea grass and Napier grass. These species have been used in crossing programmes and named varieties are commercially available from seed companies, whilst other forage species are selections from the wild and are naturally adapted to a wide range of contrasting habitats. For range grassland species, for example, to persist requires seeds able to survive in the soil seedbank for considerable periods after shedding, and/or to survive passage through the animal gut. Such characteristics require traits of (considerable) seed dormancy and/or hardseededness, which hinder the promotion of prompt germination when accessions are sown for testing, regeneration, or utilisation. Hence the promotion of seed germination can require considerable intervention, including the use of growth promoters, such as potassium nitrate and gibberellic acid, as well as seed coat removal in grasses and testa scarification in legumes and/or long test periods [21,29]. 

The major consequence of these inherent traits of forages for genebank regeneration is that as seed harvest time approaches, the seeds are at different stages of development and maturation on any one day, with the likelihood of seed being shed from plants increasing as the harvest is delayed, whilst other seeds ripen. Shedding not only reduces the numbers of seed harvested but may also cause subsequent confusion in the gene bank. Seeds of grass accessions may appear dormant to some, whereas the problem is one of “empty seeds” whereby seed were shed, but the firm seed covering structures may give the impression that the seed remains present, e.g., in *Brachiaria humidicola* [30]. Three different methods are widely used to identify and quantify empty seeds within accessions [29]: dissection (particularly of seeds that fail to germinate when tested); X-radiography; and (rarely used by genebanks to identify empty seeds but routinely used for seed cleaning) the use of a seed blower to separate by density the empty seed fraction. X-radiography can be combined with a subsequent germination test on the same seed sample and so save valuable seeds and provide a permanent image for records [29]. “Empty seeds” are not expected in forage legumes, but X-ray images can identify poorly-developed seeds and also those with insect damage.

The decision as to when to harvest forage seeds is, therefore, a compromise between the quality and uniformity of the seed and maximising their number. Consequently, many genebanks undertake the time- and labour-consuming daily sequential harvesting of ripe seeds: first, to maximise the number of seeds harvested; and second, to reduce selection and change in genetic integrity of the accession which would occur if only early- or late- flowering and maturing plants were selected.

In terms of when to harvest seed, there is no single point in developmental time across all species and all environments where seed quality (subsequent seed storage longevity) is maximal [31]. Nonetheless, harvesting when seeds are at, or almost at, harvest maturity (i.e., their moisture content is close to equilibrium with ambient relative humidity) is a reasonable solution to this dilemma because although maximal seed quality may have been attained prior to this time net seed deterioration is rarely observed on any scale before harvest maturity [31]. This tallies with forage seed production practices where the harvest is when the earliest to develop seeds or pods change colour as they mature visually, to, for example, yellow or brown, or shortly before they would be shed; and similarly in wild species more generally [6,32]. Whilst the lack of uniformity in seed maturation in forages means that the seed crop varies in the timing of harvest maturity, where direct harvesting is not possible, forage seed producers use a variety of seed harvesting approaches to reduce the consequences of variation in developmental timing (Table 3). Combing, bagging, and suction tend to be associated with multiple, sequential harvests of seed from plants where seed maturity date varies considerably—and these approaches are all used by forage plant genebanks. 

## 5. Seed Longevity

Monitoring the viability of seed of accessions in large collections regularly is costly in terms of seeds, labour and other resources, and it is important, therefore, to prioritise monitoring those accessions with brief longevity [34]. Assessing the seed storage longevity of wild species in genebanks is complex and difficult to predict as it is influenced by genotype, production environment, post-harvest handling and processing and storage conditions [7], and until recently, there was limited information on the longevity of many species [6]. Lack of data for evidence-based longevity predictions for forages led to species being classed as good or poor storers [35]. However, recent analysis of over 30 years of germination monitoring data from seeds stored in the medium-term store (seeds stored at circa 8 °C with 5% moisture content) in the genebank at ILRI allowed the calculation of seed longevity under these conditions for a range of forage genera and identified many, but not all, forage grass and legume species as having long-lived orthodox seeds [11,12]. Variation was observed in seed longevity among genera and between species of the same genus and indicated that some forage seeds have minimal dormancy and a longevity comparable to seeds of the major food crops, whilst other species showed high levels of hardseededness or dormancy that required dormancy-breaking procedures to be used in the germination test protocols.

Advice on breaking seed dormancy and promoting germination for each of 58 plant families is already published [21]. Typical successful dormancy-breaking procedures suggested therein for grasses include a germination test environment of a constant 16–21 °C or alternating temperature of 23/9 °C (12 h/12 h) for temperate accessions, or a constant 21–26 °C or alternating temperature of 33/19 °C (12 h/12 h) for tropical accessions, and/or 10^−3^ M potassium nitrate, removal of seed-covering structures, and pre-chilling at 2–6 °C for up to 8 weeks. One caution is that although (white) light can promote seed germination, the germination of some grasses can be inhibited by high light intensity: for example, in the temperate grasses *Bromus mollis* and *Bromus sterilis* [36] and the tropical grasses *Echinochloa turnerana*, *Panicum maximum* and *Brachiaria humidicola* [37]. This can be avoided by limiting the period of exposure to light each day, as well as the dose. Typical successful dormancy-breaking/germination promoting procedures suggested for legumes [21] are seed scarification and a germination test environment of a constant 11–16 °C or alternating temperature of 23/9 °C (12 h/12 h) for temperate accessions, or a constant 21–26 °C or alternating temperature of 33/19 °C (12 h/12 h) for tropical accessions. One caution is that very dry legume seeds can be sensitive to a rapid uptake of water and so may benefit from initial hydration in a moist atmosphere at 100% relative humidity. A further source of readily-available information on characteristics of seed of diverse species relevant to genebanks is the Seed Information Database (Royal Botanic Gardens Kew, http://data.kew.org/sid/) [38]. This online compilation of various types of seed information from a wide range of sources, including for seed storage behavior [39], is provided to support seed genebank operations globally by the Millennium Seed Bank Project.

Monitoring seed viability in a large collection of many individual accessions using germination tests is labour intensive, time-consuming, and depletes the amount of seeds available for each accession. Hence monitoring should be kept to a minimum, but it is nonetheless essential to determine when germination declines and thus regeneration becomes necessary to avoid the loss of that accession from the genebank. The rational determination of accession monitoring interval is important for efficient, low-cost, but effective genebank management. Analysis of historic genebank data has been applied to provide evidence-based estimates of the different monitoring intervals necessary for diverse forage species [11,12] and indicated that 15-year monitoring intervals are suitable for many long-lived forage seeds with high viability at the time of entering storage. Even in medium-term seed stores, high-quality seed of some forages need only infrequent monitoring (Table 4).

There is a virtuous circle amongst high initial accession viability, excellent storage environment, monitoring period, and regeneration period for the genetic integrity of accessions. The first two support infrequent monitoring and yet more infrequent regeneration, whilst loss in viability during storage, accession depletion through frequent monitoring, and frequent regeneration each put the genetic integrity of accessions at risk [24,40]. Moreover, accession monitoring and regeneration are expensive undertakings. It is essential to retain much larger samples for regeneration than for distribution to users if minor alleles at a low frequency in a genetically heterogeneous accession are to be conserved through cycles of storage and regeneration (infrequent if seed viability is maintained long term). One analysis suggests that, if feasible, large samples of 500 seeds be grown out to regenerate the most original seed source to maintain allelic richness within an accession containing many alleles at low frequency [24].

## 6. Cost Efficiency of Managing Wild Species

The biological differences between crop species on the one hand and forage and wild species on the other are reflected in the costs of management and conservation in genebanks. There is limited data available on the actual costs of conservation other than from the genebanks of the centres of the Consultative Group for International Agricultural Research (CGIAR) [41,42]. Using these data as an example, the cost of conservation and management per sample in CIAT in 2000 was more than double for forages than for beans [41]. Costs also differ between locations even for the conservation of the same species. Studies in 2006 and 2009 calculated the costs of acquisition, characterisation, safety duplication, medium and long term storage, germination and seed health monitoring, regeneration, seed processing, information management, distribution and general management and concluded that the cost of forage germplasm conserved in CIAT was $315 per accession, whilst in ILRI these estimates ranged from $125–242 per accession, depending on the type of forage [42]. Such cost comparisons must account for the costs of operating in specific locations worldwide, as well as those incurred by different processes and procedures, and each Centre’s prioritisation of species and activities. The reproductive biology of the species, which determines the type of conservation and regeneration procedures, and the quality of management attained in the genebank, contributed most to the variation in the costs of genebank operations [42]. Crops with orthodox seeds had the lowest costs of conservation, whilst vegetatively propagated crops, trees and wild relatives had higher costs.

The size of the collection also contributes to the costs of management. There is a marginal cost to adding an additional accession into a seed genebank with sufficient space to accommodate the seeds, but the benefit to cost ratio is high. The basic questions that genebank managers ask are what is the value of adding additional accessions, which germplasm should genebanks collect and store, and when is the collection large enough and contains the genetic traits demanded by users. The probability of finding accessions of value for users depends on the gene frequency of the trait that is being sought, how many accessions are tested and whether accessions are selected at random or selected based on prior knowledge on where to look for specific genes. Economic principles can be applied to modelling the value of accessions [43]. Sampling more accessions provides a higher probability of finding genes in low frequencies within the accessions but comes at a cost. For rare genes with a probability of 0.01 of presence in accessions, sampling 200 seeds gives an 87% chance of the gene being present in the sample. There is a marginal or incremental probability of finding the gene in the population, and the probability reduces as more accessions are added because if the gene is rare, the marginal value of adding an accession is low because it is difficult to find the gene. If the gene is common, the marginal value of adding accessions is also low because it has probably already been located. The marginal value of adding more accessions is high for intermediate ranges of gene scarcity [43,44]. Economically this supports keeping collections of wild species and forages relatively small, in terms of the number of accessions within a single species, because of their lower probability for use and high costs of conservation resulting from their reproductive biology and conservation challenges.

Cost–benefit analysis could be used to determine if the benefits from using the germplasm in forage development that has been, or could, in future, be realised, justify the conservation costs. However, this would require future germplasm utilisation to be quantified, which is very difficult to do. The value of the germplasm within genebanks lies in the future—when it will be used to transfer useful traits to forage cultivars. The option value of having the germplasm available to respond to as yet unidentified future needs, such as changing feed needs due to climate change or intensifying livestock production systems, and the existence value that society derives from knowing that something exists and will be available for future needs may be more powerful economic drivers of conservation of forage diversity than the cost–benefit analyses that can be done on its actual use [45]. In reality, most genebanks have fixed budgets, and so the management priority is to apply those resources to the best effect. 

One example is the relative cost and risk of collecting forages and conserving ex situ in seed or field genebanks or maintaining them in situ in conservation areas within the extensive grasslands where they originated and continue to evolve and adapt. In situ conservation is a good alternative for forage species that are well protected in the wild, provided there is no evidence of a future threat or of historic genetic erosion to these forage genotypes. A good example of this is in sub-Saharan Africa, where many of the indigenous forages are found in well-protected rangelands in national parks [46]. On the other hand, ex situ conservation of forage germplasm is an essential precaution where over-grazing and other poor land management practices occur; it also makes the germplasm more accessible to users.

## 7. Policy Issues

The legal framework for crop collections is the multilateral system of the International Treaty on Plant Genetic Resources for Food and Agriculture (ITPGRFA) [47]. This covers the major crops where countries have high interdependence on germplasm for food security, allowing them to be freely exchanged under a standard material transfer agreement (SMTA) to be used for food and agriculture with benefit-sharing provisions based on their commercial use. Germplasm flows through facilitated access under the ITPGRFA from the genebanks of the CGIAR Centres are well documented [48,49] with 4 million samples of germplasm made available under the SMTA from 2007–2016 to national partners and breeders, representing 93% of reported global distributions under the multilateral system [49].

The list of crops covered by the ITPGRFA includes some temperate forages and crop wild relatives that are also used as forages, but not many of the common and important tropical forage species in Table 1 of this review. Expanding collections with new germplasm will need to be done under bilateral agreements on access and benefit-sharing under the terms of the Nagoya Protocol. Reaching bilateral agreements on access and benefit-sharing under the Nagoya Protocol will be complicated and, in some cases, may not be possible in the short term as countries reform their national laws on access [50].

Whilst the economic cost–benefit analysis of conserving large ex situ collections of forages combined with the option to conserve forages in secure conservation areas in national parks indicates that there are other more cost-effective options for the conservation of forage diversity in many cases, the forage germplasm currently held in ex situ collections may well be the more accessible source for research and forage development because it avoids the need to collect and to negotiate conditions of access and benefit-sharing to new germplasm. The latter may take time, and so it is important that the germplasm already collected is properly managed and safeguarded in ex situ genebanks and continues to be made available to users.

## 8. Conclusions

Although collections in genebanks are vulnerable to loss of diversity [51] and the conservation of forage in ex situ collections has many challenges as outlined in this review, seed storage remains the most cost-effective and efficient method for their conservation and sustainable use for the immediate future. Ex situ conservation of forage germplasm must be linked to use in forage development to realise the potential benefits from the high costs of conservation. Unlike crops, where genebank accessions are used in breeding programmes to combine with other genotypes, many forage accessions are selected for direct use based on their adaptation, productivity and nutritional quality and released as cultivars due to the limited number of forage breeding programmes [4] and time and cost required to screen large numbers of accessions for useful traits [5]. To use wild species as forages, the challenges faced in seed dormancy, germination and harvesting for seed production of these species will need to be addressed methodically and research carried out to improve ease of cultivation and support adoption. 

There are fundamental questions on which germplasm genebanks should collect and store, optimum size of the collection and how much diversity is sufficient to meet user needs remain open for forages. The answer depends on what types of forages are required for future sustainable livestock systems. Climate and environmental issues are shaping the perception of consuming livestock products in western diets, and people are being urged to reduce meat consumption. Yet in the developing world, many children survive on diets deficient in proteins and animal products and require milk and meat in their diets for cognitive development [52]. Livestock production systems are intensifying in the tropics with an increase in crossbred dairy cows with higher feed requirements. Natural pastureland is reducing in area as grassland and forest are converted for cropping, whilst climates are tending to dry and warm, pushing forage production to marginal land or planted as part of intensified crop-livestock production systems. The intensification of dairy systems through the use of higher quality feed in East Africa is predicted to increase milk yields without increasing greenhouse gas emissions, addressing both productivity and environmental issues [53]. This intensification is leading to the production of more crop residues and by-products for feed and, as a result, the need for higher quality protein-rich forages to supplement these lower quality residues. Additionally, in an effort to reduce greenhouse gas emissions, forage producers could reduce the use of inorganic nitrogen fertilisers replacing them with forage legumes, capable of symbiotic nitrogen fixation, and use more perennial forage shrubs and trees to reduce carbon loss from soils from cultivation. Hence, it is likely that the forages of the future will be developed from accessions of forage legumes and fodder tree species from ex situ collections to meet these needs. Given the anticipated continued demand for forage germplasm, challenges in their management will be reduced through research to support their conservation and availability for forage development. 

## Figures and Tables

**Table 1:** Common tropical and sub-tropical forage species and climate zone suitability for cultivation.

Forage Species	Climatic Zone of Cultivation				
	Arid	Semi-Arid	Sub-Humid	Humid	Highland
**Herbaceous legumes**					
*Aeschynomene americana*			√		
*Alysicarpus glumaceus*		√			
*Arachis pintoi*			√	√	
*Calopogonium mucunoides*			√	√	
*Centrosema acutifolium*			√	√	
*Centrosema brasilianum*		√			
*Centrosema macrocarpum*				√	
*Centrosema pascuorum*		√	√		
*Centrosema pubescens*			√	√	
*Chamaecrista rotundifolia*		√	√		
*Clitoria ternatea*	√	√	√		
*Desmodium intortum*			√		√
*Desmodium uncinatum*			√		
*Lablab purpureus*	√	√	√		
*Macroptilium atropurpureum*		√	√		
*Macrotyloma axillare*			√		√
*Medicago sativa*					√
*Neonotonia wightii*		√	√		
*Pueraria phaseoloides*			√	√	
*Rhynchosia minima*		√			
*Stylosanthes fruticosa*	√	√			
*Stylosanthes guianensis*		√	√	√	
*Stylosanthes hamata*	√	√	√	√	
*Stylosanthes scabra*		√	√	√	
*Stylosanthes seabrana*			√	√	
*Teramnus labialis*			√		
*Trifolium decorum*					√
*Trifolium rueppellianum*					√
*Trifolium pratense*					√
*Trifolium quartinianum*					√
*Trifolium repens*					√
*Trifolium semipilosum*					√
*Trifolium steudneri*					√
*Trifolium tembense*					√
*Vicia villosa*					√
*Vicia sativa*					√
*Vigna unguiculata*	√	√	√		
*Zornia glabra*		√	√		
*Zornia latifolia*		√	√		
**Fodder trees**					
*Cajanus cajan*		√	√		
*Calliandra calothyrsus*			√		
*Cytisus proliferus*					√
*Desmanthus virgatus*			√	√	
*Faidherbia albida*		√			
*Gliricidia sepium*		√	√	√	
*Leucaena diversifolia*					√
*Leucaena leucocephala*		√	√	√	
*Leucaena pallida*					√
*Leucaena revoluta*					√
*Sesbania sesban*		√	√		√
**Grasses**					
*Avena sativa*					√
*Bothriochloa pertusa*			√		
*Brachiaria decumbens*			√	√	
*Cenchrus ciliaris*		√	√		
*Chloris gayana*		√	√		
*Cynodon dactylon*		√	√		
*Melinis minutiflora*			√		
*Panicum coloratum*		√	√		√
*Panicum maximum*			√	√	
*Paspalum dilatatum*			√		
*Paspalum plicatulum*			√		√
*Pennisetum clandestinum*					√
*Pennisetum purpureum*			√	√	
*Setaria sphacelata*			√	√	
*Sorghum almum*		√	√		
*Urochloa mosambicensis*			√	√	

Zone definitions; Arid zone-100–500 mm rainfall, 0–180 growing days per annum; Semi-arid zone-600–1000 mm rainfall, 0–180 growing days per annum; Sub-humid zone-1000–1500 mm rainfall, 180–270 growing days per annum; Humid zone- >1500 mm rainfall, >270 growing days per annum; Highland zone- >1500 m altitude.

**Table 2:** Examples of information sources (reference numbers shown) on methods of seed production; dormancy (and dormancy-breaking methods), germination (germination testing and promotion for accession monitoring), and seed survival in genebanks (seed survival periods of different genera of forage grasses and forage legumes in genebanks).

Family/Genus	Seed Production	Dormancy/Germination	Seed Survival in Genebanks
Fabaceae			
*Acacia*		[8,21]	[9,11]
*Aeschynomene*	[13]		[11]
*Albizia*			[11]
*Alysicarpus*	[13,15]		[11]
*Argyrolobium*			[11]
*Astragalus*	[15,17]	[8,21]	
*Cajanus*			[11]
*Calopogonium*	[13]	[8,21]	[11]
*Canavalia*			[11]
*Cassia*	[13]		[11]
*Centrosema*	[13,17,19]	[8,21]	[11]
*Chamaecrista*			[11]
*Clitoria*			[11]
*Coronilla*	[15,17]	[8,21]	
*Crotalaria*	[15]	[8,21]	[11]
*Desmanthus*			[11]
*Desmodium*	[13]	[8,21]	[11]
*Entada*			[11]
*Erythrina*			[11]
*Faidherbia*			[11]
*Galactia*			[11]
*Gliricidia*			[11]
*Glycine*		[8,21]	[9,11]
*Indigofera*	[15]	[21]	[11]
*Lablab*	[13]	[8,21]	[11]
*Lathyrus*	[15]	[8,21]	[9,11]
*Lespedeza*	[15,17,19]	[8,21]	[9]
*Leucaena*	[13,17]	[21]	[11]
*Lotononis*	[13]	[8,21]	[11]
*Lotus*	[15,17,19]	[8,21]	[9,11]
*Lupinus*	[15]	[8,21]	[9,11]
*Macroptilium*	[17,19]	[8,21]	[11]
*Macrotyloma*			[11]
*Medicago*	[15,17,19]	[8,21]	[9,11]
*Melilotus*	[15,17,19]	[8,21]	[9,11]
*Mucuna*		[8,21]	[11]
*Neonotonia*			[11]
*Onobrychis*	[15,17,19]	[8,21]	[9]
*Ornithopus*	[17,19]	[8,21]	[11]
*Phaseolus*		[8,21]	[9,11]
*Pisum*	[15]	[8,21]	[9,11]
*Prosopis*			[11]
*Pseudarthria*			[11]
*Psophocarpus*		[8,21]	[11]
*Pueraria*	[15]	[8,21]	
*Rhynchosia*			[11]
*Senna*			[9,11]
*Sesbania*		[21]	[11]
*Stizolobium*	[15]		
*Stylosanthes*	[13,17,19]	[8,21]	[11]
*Tephrosia*		[8,21]	[11]
*Teramnus*			[11]
*Trifolium*	[13,15,17,19]	[8,21]	[9,10,11]
*Vicia*	[15,17,19]	[8,21]	[9,11]
*Vigna*	[15]	[8,21]	[9,11]
*Zornia*	[13]		[11]
Poaceae			
*Aegilops*		[21]	
*Agropyron*	[15,17,19]	[8,21]	[9,12]
*Agrostis*	[15,17]	[8,21]	[9,10]
*Alopecurus*	[17,19]	[8]	
*Andropogon*	[13,15,17,19]	[8,21]	[12]
*Arrhenatherum*	[15,17,19]	[8]	
*Aristida*		[21]	[12]
*Avena*		[8,21]	[9,12]
*Axonopus*	[15]	[8]	
*Bothriochloa*	[13]	[8,21]	[12]
*Bouteloua*	[15,17]	[8,21]	
*Brachiaria*	[13,17,19]	[8,21]	[12]
*Bromus*	[15,17,19]	[8,21]	[9,12]
*Buchloe*	[15]	[8]	
*Cenchrus*	[13,17]	[8]	[12]
*Chloris*	[13,15,19]	[8,21]	[12]
*Cymbogon*		[21]	
*Cynodon*	[15]	[8,21]	[12]
*Dactylis*	[15,17,19]	[8,21]	[9,10,12]
*Digitaria*		[8,21]	[12]
*Echinochloa*		[8,21]	[9,12]
*Eleusine*		[8,21]	[9,12]
*Elymus*	[15,17,19]	[8]	[9,12]
*Eragrostis*	[13,15,17]	[8,21]	[9,12]
*Eremochloa*	[15]		
*Festuca*	[15,19]	[8,21]	[9,10,12]
*Hordeum*	[15]	[8,21]	[9,12]
*Hyparrhenia*	[13]		
*Lolium*	[15,17]	[8,21]	[9,12]
*Melinis*	[17]	[8]	[12]
*Oryzopsis*	[15]	[8,21]	
*Panicum*	[13,15,17]	[8,21]	[9,12]
*Paspalum*	[13,15,17]	[8,21]	[12]
*Pennisetum*	[13,15,17]	[8,21]	[9,12]
*Phalaris*	[15,17]	[8,21]	[12]
*Phleum*	[15,17,19]	[8,21]	[9,10,12]
*Poa*	[15,17]	[8,21]	[9,10]
*Setaria*	[13,17,19]	[8,21]	[9,12]
*Sorghastrum*	[15]	[8,21]	
*Sorghum*	[17,19]	[8,21]	[9,12]
*Sporobolus*	[15]		[9]
*Stipa*	[15]	[21]	
*Themeda*		[21]	
*Tripsacum*	[15]		
x*Triticosecale*		[8,21]	[12]
*Urochloa*	[13,17]	[8]	[12]

**Table 3:** Seed harvesting techniques for accessions which show considerable plant-to-plant variation in the timing of seed maturity.

Detail	Swathing	Swathing and Sweating	Desiccation	Combing	Bagging	Suction
Description	Cutting the seed crop and leaving in a loose swath to dry for later mechanical harvesting	Heaping or sheathing seed heads for about 3 days before threshing to raise temperature	Spraying chemical desiccant on the crop to accelerate the drying of less-mature seed	Running a hand over the seed heads or shaking seed heads gently into a bag	Covering seed heads or pods with a net, cloth or paper bag	Vacuuming mature seeds from the plant or ground under the plant
Advantages	Maturation of later-developing seeds as moisture uptake ends and swath dries; capture of more mature seeds in the swath if they shatter; low labour requirement	Aids maturation and abscission [33]	Enables direct harvesting of dry seeds; low labour requirement	Only mature seeds are collected	Captures seeds that shatter or dehisce	Immature seeds remain on the plant to mature and ripe seeds are not lost by shattering
Disadvantages	Some seeds will be lost from the swath; specialist equipment	Immature seeds may also abscise; prolonged treatment may damage seeds; specialist equipment	Cost and risk of desiccant; specialist equipment	Labour intensive; repeated, frequent combing required	Labour intensive; fungal contamination in bags during rains	Labour intensive; weed seeds and debris can be collected from the ground
Examples	*Stylosanthes* species; temperate grasses	*Panicum maximum;* tropical grasses	*Vicia faba; Avena sativa*	*Vicia villosa*	*Brachiaria* species	*Stylosanthes* species; *Buchloe dactyloides* [17]

**Table 4:** List of forage legume species with long-lived seeds under medium-term storage (circa 8 °C with 5% moisture content) at ILRI for which monitoring intervals of 15 years for high-quality seed accessions under such storage conditions are proposed.

Genus	Specific Epithet of the Species
*Acacia*	*angustissima, boliviana*
*Alysicarpus*	*ferrugineus, glumaceus, longifolius, monilifer, ovalifolius, rugosus, vaginalis*
*Calapogonium*	*mucunoides*
*Chamaecrista*	*mimosoides, nigricans, pilosa, rotundifolia*
*Desmanthus*	*acuminatus, covillei, leptophyllus, pubescens, tatuhyensis, virgatus*
*Desmodium*	*adscendens, barbatum, cinereum, dichotomum, discolor, distortum, incanum, intortum, salicifolium, sandwicense, tortuosum, uncinatum, velutinum*
*Indigofera*	*arrecta, brevicalyx, colutea, cryptantha, hirsuta, hochstetteri, spicata, suffruticosa*
*Leucaena*	*diversifolia, leucocephala, pallida, pulverulenta, shannonii, trichandra, trichodes*
*Lupinus*	*albus, angustifolius, luteus, mutabilis*
*Macroptilium*	*atropurpureum, bracteatum, lathyroides*
*Macrotyloma*	*africanum, axillare, daltonii, uniflorum*
*Medicago*	*lupulina, minima, polymorpha, sativa, scutellata, truncatula*
*Melilotus*	*albus, officinalis*
*Pisum*	*sativum*
*Stylosanthes*	*calcicola, capitata, fruticosa, guianensis, hamata, humilis, scabra*
*Tephrosia*	*bracteolata, noctiflora, pumila, purpurea, villosa*
*Teramnus*	*labialis, repens, uncinatus*
*Trifolium*	*baccarinii, bilineatum, burchellianum, cryptopodium, decorum, masaiense, mattirolianum, multinerve, polystachyum, pratense, quartinianum, repens, resupinatum, rueppellianum, semipilosum, simense, steudneri, tembense*
*Vigna*	*luteola, oblongifolia, parkeri, peduncularis, racemosa, radiata, unguiculata, vexillata*
*Zornia*	*glabra, latifolia*
